# Acceptability of the Medication Event Reminder Monitor for Promoting Adherence to Multidrug-Resistant Tuberculosis Therapy in Two Indian Cities: Qualitative Study of Patients and Health Care Providers

**DOI:** 10.2196/23294

**Published:** 2021-06-10

**Authors:** Beena E Thomas, J Vignesh Kumar, Murugesan Periyasamy, Amit Subhash Khandewale, J Hephzibah Mercy, E Michael Raj, S Kokila, Apurva Shashikant Walgude, Gunjan Rahul Gaurkhede, Jagannath Dattatraya Kumbhar, Senthanro Ovung, Mariyamma Paul, B Sathyan Rajkumar, Ramnath Subbaraman

**Affiliations:** 1 Department of Social and Behavioural Research ICMR-National Institute for Research in Tuberculosis Chennai India; 2 Department of Public Health and Community Medicine Tufts University School of Medicine Boston, MA United States; 3 Center for Global Public Health Tufts University School of Medicine Boston, MA United States; 4 Division of Geographic Medicine and Infectious Diseases Tufts Medical Center Boston, MA United States

**Keywords:** tuberculosis, drug-resistant, medication adherence, mHealth, digital adherence technologies, India

## Abstract

**Background:**

Patients with multidrug-resistant tuberculosis (MDR-TB) face challenges adhering to medications, given that treatment is prolonged and has a high rate of adverse effects. The Medication Event Reminder Monitor (MERM) is a digital pillbox that provides pill-taking reminders and facilitates the remote monitoring of medication adherence.

**Objective:**

This study aims to assess the MERM’s acceptability to patients and health care providers (HCPs) during pilot implementation in India’s public sector MDR-TB program.

**Methods:**

From October 2017 to September 2018, we conducted qualitative interviews with patients who were undergoing MDR-TB therapy and were being monitored with the MERM and HCPs in the government program in Chennai and Mumbai. Interview transcripts were independently coded by 2 researchers and analyzed to identify the emergent themes. We organized findings by using the Unified Theory of Acceptance and Use of Technology (UTAUT), which outlines 4 constructs that predict technology acceptance—performance expectancy, effort expectancy, social influence, and facilitating conditions.

**Results:**

We interviewed 65 patients with MDR-TB and 10 HCPs. In patient interviews, greater acceptance of the MERM was related to perceptions that the audible and visual reminders improved medication adherence and that remote monitoring reduced the frequency of clinic visits (performance expectancy), that the device’s organization and labeling of medications made it easier to take them correctly (effort expectancy), that the device facilitated positive family involvement in the patient’s care (social influences), and that remote monitoring made patients feel more *cared for* by the health system (facilitating conditions). Lower patient acceptance was related to problems with the durability of the MERM’s cardboard construction and difficulties with portability and storage because of its large size (effort expectancy), concerns regarding stigma and the disclosure of patients’ MDR-TB diagnoses (social influences), and the incorrect understanding of the MERM because of suboptimal counseling (facilitating conditions). In their interviews, HCPs reported that MERM implementation resulted in fewer in-person interactions with patients and thus allowed HCPs to dedicate more time to other tasks, which improved job satisfaction.

**Conclusions:**

Several features of the MERM support its acceptability among patients with MDR-TB and HCPs, and some barriers to patient use could be addressed by improving the design of the device. However, some barriers, such as disease-related stigma, are more difficult to modify and may limit use of the MERM among some patients with MDR-TB. Further research is needed to assess the accuracy of MERM for measuring adherence, its effectiveness for improving treatment outcomes, and patients’ sustained use of the device in larger scale implementation.

## Introduction

### Background

Multidrug-resistant tuberculosis (MDR-TB) is a major global challenge to tuberculosis (TB) control. In 2018, approximately 484,000 people worldwide were estimated to have developed MDR-TB, including approximately 130,000 people in India [1]. Despite considerable advances in therapy in the last decade, treatment outcomes remain poor for individuals with MDR-TB, with treatment success rates of 56% worldwide and 48% in India for the 2017 patient cohort [1]. Although some of the variability in treatment outcomes may be attributable to the composition of the patient’s drug regimen [2], suboptimal adherence to medications may be another critical problem contributing to poor MDR-TB treatment outcomes.

Successful adherence for diseases with a prolonged treatment course, such as MDR-TB, requires a high level of dosing implementation (ie, taking a medication dose on a given day) and persistence (ie, taking medications for the entire duration of therapy [3]). Factors contributing to nonadherence are complex and include therapy-related (eg, toxicities [4]), psychosocial (eg, alcohol use [5], depression [6], and stigma [7]), structural (eg, distance from clinics and medication costs [8,9]), and health system–related challenges (eg, poor user experience with the health system). Patients with MDR-TB face particularly high levels of drug toxicity [4] and psychosocial barriers, including depression, substance use disorders, stigma, and discrimination [10]. These challenges may lead to poor outcomes and increased transmission of drug-resistant strains.

As such, there is an urgent need for new strategies to improve medication adherence in patients with MDR-TB. Many TB programs have historically used directly observed therapy (DOT) to monitor adherence; however, recent studies have questioned the efficacy of this strategy for improving clinical outcomes [11-13] and raised concerns that DOT adversely affects patient autonomy [14,15]. Limited autonomy with DOT may be greater for patients receiving MDR-TB treatment, given the prolonged course of therapy required. In addition, recent recommendations favoring the use of regimens containing only oral medications may decrease the required frequency of clinic visits for patients with MDR-TB if they are allowed to take therapy without in-person observation [16].

In recent years, driven by the global expansion of cellular networks, there has been a growing use of digital adherence technologies (DATs) as alternative approaches for monitoring adherence to TB medications [3]. These technologies, which include cellphones, digital pillboxes, and ingestion sensors, have the potential to improve clinical outcomes via multiple pathways [3]. Although there are numerous DATs aimed at addressing nonadherence in patients with drug-susceptible TB [3], few have attempted to address the more complex medication regimens taken by patients with MDR-TB [17].

The Medication Event Reminder Monitor (MERM) is a digital pillbox that has been designed to monitor MDR-TB treatment in resource-constrained settings by using relatively affordable evriMED technology produced by Wisepill Technologies. This system is designed to be used with multiple blister-packaged medications in MDR-TB regimens, incorporates visual and audible reminders for daily dosing and refills, compiles dosing histories by capturing data on pillbox opening as a proxy for dose ingestion, and transmits these data to a server so that health care providers (HCPs) can remotely visualize patients’ dosing histories. By providing near real-time adherence data, the MERM may facilitate the identification of high-risk patients and prompt early intervention by HCPs to reduce nonadherence. When compared with facility-based DOT, in which patients travel to clinics to be observed taking medications, monitoring by using the MERM may reduce the required frequency of patient visits to TB clinics.

Pilot studies of digital pillboxes conducted in Uganda and China with patients with drug-susceptible TB and in South Africa with patients with MDR-TB have shown these devices to have relatively high acceptability [17-19]. A cluster randomized trial conducted in China with patients with drug-susceptible TB found digital pillboxes to be effective in reducing the percentage of patient months with high nonadherence [20]. However, subsequent studies on the large-scale implementation of these digital pillboxes in China have revealed challenges in their uptake. For example, after accounting for patients who were not eligible to use these pillboxes, refused to use them, withdrew from using them early in treatment, or were shifted to monitoring with DOT, only approximately 49% and 39% of patients with TB used digital pillboxes in a sustained manner in a single province [21] and 30 counties [22], respectively, in China. In addition, a study of the MERM conducted in Vietnam with patients with drug-susceptible TB found that only approximately half of the patients used the device as intended, with many separating the time when the pillbox was opened from the time that doses were ingested, because of concerns about the device’s portability [23]. These existing studies evaluating the use of digital pillboxes reveal variability in patient acceptance in different contexts and highlight a relative paucity of data on the use of these devices for patients with MDR-TB.

### Objectives

In this study, based on qualitative interviews with patients and HCPs, we evaluated the MERM’s ability to monitor adherence to MDR-TB therapy during pilot rollout in the government’s National TB Elimination Program (NTEP) in 2 Indian cities. Although this novel strategy has potential advantages, previous research has not been conducted in India to evaluate whether patients will accept and use the MERM, to identify potential modifiable and nonmodifiable barriers to its acceptability, and to understand how its implementation might impact HCP work efficiency and quality of care. Understanding the acceptability of MERM during pilot implementation is also important because recent studies of other DATs in India suggest that suboptimal acceptability and use by patients could reduce the accuracy of these technologies for measuring adherence [24], which might in turn greatly reduce the value of real-time adherence data for guiding interventions. We analyze our findings using the Unified Theory of Acceptance and Use of Technology (UTAUT), a framework that synthesizes constructs that predict engagement with novel technologies [25,26].

## Methods

### Ethical Approvals

This protocol received approval from the Indian Council of Medical Research-National Institute for Research in TB Institutional Ethics Committee (FWA00005104) on March 7, 2017. The study was approved by the Brigham and Women’s Hospital Institutional Review Board (FWA00000484) on January 31, 2017, and the Tufts Health Sciences Institutional Review Board (FWA00004517) on June 6, 2018. Written informed consent was obtained from all the participants.

### Study Setting

This study was conducted in 2 Indian cities with a high TB burden in the general population [27,28]: Chennai (estimated population of 7.1 million in metropolitan area), in the southern Indian state of Tamil Nadu, and Mumbai (estimated population of 18.4 million in the metropolitan area), in the western Indian state of Maharashtra. Mumbai in particular has one of the world’s most severe urban epidemics of drug-resistant TB [29-31]. All patients in Chennai spoke Tamil, and all patients in Mumbai spoke Hindi or Marathi. Consistent with the broader population of patients with TB who seek government care in India, more than half of whom earn less than US $2 a day of income [32], patients with MDR-TB in these 2 cities come from socioeconomically disadvantaged backgrounds.

### MERM Implementation

**Figure 1:**
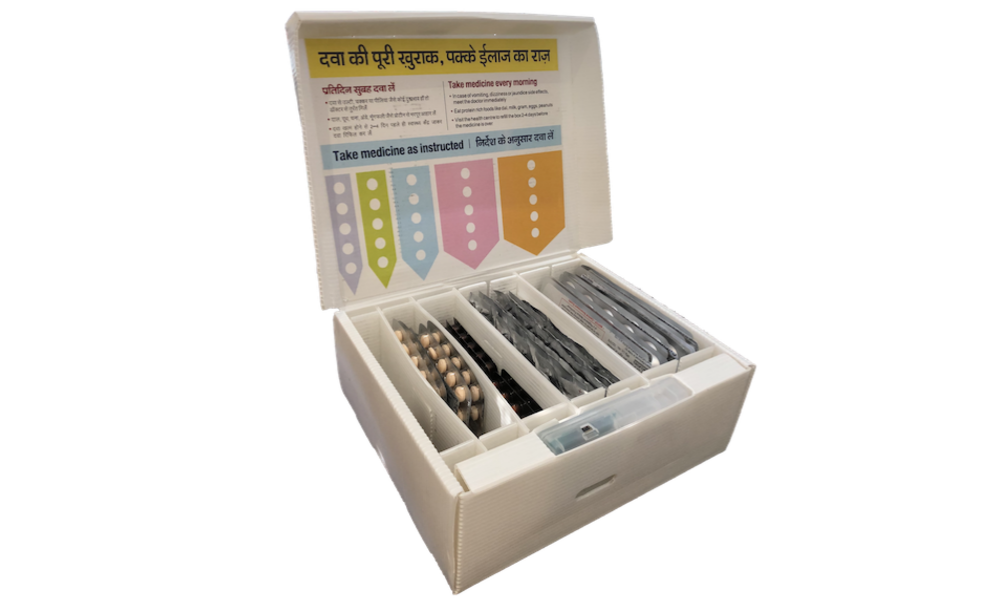
The Medication Event and Reminder Monitor in a cardboard version used for the initial rollout among patients with multidrug-resistant tuberculosis in India. The device includes partitions for organizing medications, medication labels inside the box lid, and a digital module that provides reminders and captures adherence data. This cardboard version was provided by Wisepill Technologies.

Medications are dispensed in blister packs, and each drug is placed in a different partitioned compartment within the MERM, which facilitates storage and organization of the multiple medications that comprise MDR-TB regimens (Figure 1). In India’s pilot implementation, the container and internal partitions were made of cardboard. The device was provided to patients at different time points in the continuation phase of therapy when injectable agents had generally been discontinued and patients were only taking oral medications. At the time of our study, most patients with MDR-TB in India’s NTEP were placed on a standardized drug regimen for a treatment course lasting 24 to 27 months, with the continuation phase consisting of levofloxacin, ethionamide, cycloserine, and ethambutol taken once daily [33]. Patients who were provided the MERM subsequently used it for the remaining duration of treatment.

The MERM was programmed to provide audible and visual reminders to take medications at a specific time each day, per patient and HCP preference. The visual reminder consisted of a blinking green light corresponding to a label encouraging the patient to take a dose; separate yellow and red lights blink to alert patients about the need to refill medications and replace the MERM’s battery, respectively. The audio reminder consisted of a ringing sound that occurred at the same time as the visual dose-taking reminder.

The device contained a removable battery-powered module. Triggered by a magnetic sensor, this module captures and stores data each time the container is opened as a proxy for medication ingestion. These data on patient engagement with the MERM were transmitted every 72 hours using cellular networks and recorded on a computer server. HCPs could log into an app on a mobile device or a website, where each patient’s adherence history was presented as a color-coded calendar in which green suggested that the MERM was opened on a given day (suggesting dose ingestion), whereas red suggested that the device was not opened (suggesting that the dose was not ingested).

These dosing histories were meant to help HCPs have individualized discussions with patients regarding their adherence. In addition, a series of probable missed doses would result in automated SMS text messaging notifications prompting HCPs to intervene with these patients, who might be at a higher risk for unfavorable outcomes.

### Recruitment of Study Participants and Collection of Qualitative Data

Interviews were conducted by 3 field researchers in Mumbai (1 man and 2 women) and 3 field researchers in Chennai (2 men and 1 woman), all of whom had a master’s degree in social work or another social science field who underwent a 2-day training in qualitative interviews at the National Institute for Research in TB in Chennai. Study participants included patients with MDR-TB and HCPs. We use the term *MDR-TB* to describe patients with confirmed resistance to isoniazid and rifampin as well as individuals who were diagnosed as having rifampin-resistant TB using Xpert MTB/RIF (Cepheid, Inc) because patients with rifampin-resistant TB in India are treated as having likely MDR-TB. Data collection was conducted a few months after the MERM was introduced in Mumbai and Chennai for monitoring patients with MDR-TB from October 2017 to September 2018. Before the rollout of the MERM, HCPs were extensively trained on the appropriate use of the MERM. HCPs dispensed medication refills in the MERM for patients with MDR-TB in the continuation phase of therapy after any injectable agents (eg, kanamycin) were discontinued.

Field researchers met patients at MDR-TB clinics, where patients were screened for inclusion in the study. At these clinics, HCPs were recruited for the study, including health visitors (individuals with at least a high school level of education who monitor therapy), senior treatment supervisors (individuals with at least a high school level of education who supervise health visitors), medical officers (doctors with an MBBS or higher degree), and district TB officers (doctors who supervise TB care across a district).

For patients, an unannounced home visit was made at least 3 weeks after enrollment into the study to conduct the qualitative in-depth interviews regarding the MERM, which lasted about 45 minutes. A pill count was also conducted to better understand the patients’ adherence to medications. We ensured a minimum of 3 weeks lapsed between when a patient was consented to the study and when the unannounced home visit was conducted. This time gap minimized the impact of temporary changes in medication adherence that may result from the patient knowing that he or she will be visited as part of the study (ie, the Hawthorne effect). For HCPs, interviews lasted about 30-45 minutes and were conducted in a private space in the TB clinic.

To ensure uniformity in data collection, separate patient and HCP interview guides, each consisting of open-ended and semistructured questions with follow-up probes, were used to conduct the interviews. Examples of questions from the patient interview guide are provided in Multimedia Appendix 1. The interview questions had the goal of assessing key constructs in the UTAUT framework. Interviews were conducted in Tamil, Hindi, or Marathi and audio recorded. They were later transcribed and translated to produce deidentified English language transcripts. To ensure translation accuracy, one-fourth of the English transcripts were evaluated against the original audio recordings for correctness and completeness.

### Analytical Framework: UTAUT

The UTAUT integrates constructs from previous literature on technology acceptance into a single framework [25,26]. Of these constructs, 3—performance expectancy, effort expectancy, and social influences—help to predict the intention of individuals to use a technology, which is necessary, but not sufficient, to result in actual use. Performance expectancy refers to the perceived usefulness of technology for users. For example, for patients with MDR-TB, this may refer to the extent to which the MERM is perceived to improve their medical care, whereas for HCPs, it may refer to the extent to which it is perceived to improve the quality or efficiency of their work. Effort expectancy refers to the ease of using the technology. For patients, this may refer to the effort required to correctly understand the different functions of the digital pillbox (eg, storage function and reminders), whereas for HCPs, this may refer to the effort required to use and understand the web-based adherence dashboard and SMS text messaging reminders that notify HCPs about nonadherent patients. Social influences refer to how other individuals may influence a person’s acceptance and use of technology. For patients, this may include family members or community residents, whereas for HCPs, this may include coworkers. Of these 3 constructs, performance expectancy has the strongest influence on the intention to use a technology [25].

Facilitating conditions, the fourth construct in the UTAUT, is thought to directly affect the actual use of a new technology. Facilitating conditions comprise the underlying infrastructure to enable the use of a technology. For patients with MDR-TB, we interpreted this broadly to include factors in the TB program, such as the quality of counseling regarding the MERM provided to patients and any outreach to patients by HCPs that might have been prompted by adherence data from the MERM. For HCPs, we interpret this to include the quality of training they received before the rollout of the MERM and any higher-level support they received during the rollout process.

### Analysis of Qualitative Data

Transcribed interviews were coded using a thematic approach and analyzed using Dedoose software (version 8.0.35; SocioCultural Research Consultants, LLC). The study team first identified possible codes (ie, themes) related to the central research question from the data collected by using the qualitative interview guides as a foundation and the UTAUT as an organizing framework. The transcripts were then independently coded by 2 researchers for relevant themes using descriptive content analysis. In parallel, the researchers tracked new codes that were added to the coding scheme to describe unexpected themes that emerged. The two researchers frequently met to reconcile inconsistencies in the application of codes and to ensure that emergent codes were added to the coding scheme. Because all coding differences were reconciled by consensus, we did not assess the interrater reliability between the coders.

We analyzed the data to identify emergent themes that could influence the acceptability and use of MERM. Emergent themes were organized within the 4 UTAUT constructs, and illustrative quotations were selected for each theme. In reporting our findings, we follow the principles of qualitative research by avoiding the quantification of codes (or themes) from our data [34]. In our findings, we report not only common themes (ie, those that emerged most frequently) but also salient themes (ie, themes reported by a minority that are still important).

We also specifically did not classify each patient based on whether they reported a high or low acceptance or use of the MERM. In contrast, we focus on reporting specific features of the MERM that were associated with higher or lower acceptance of the device because individual patients might find some components to be acceptable while simultaneously finding other components to be unacceptable. For example, a patient might appreciate the MERM’s organization of medications but, at the same time, have concerns about the audible reminder because of fear that it could lead to the disclosure of her or his MDR-TB diagnosis. In addition, there is often individual variation in whether patients accept a particular technology [35]. As such, we avoid making a blanket declaration that the device is either *acceptable* or *unacceptable* to the larger patient population with MDR-TB in India.

## Results

### Characteristics of Study Participants

We interviewed 65 patients with MDR-TB, for whom the median age was 33 years (range 18-75 years).

Home visits were conducted for a median of 5 weeks (range 3-8 weeks) after the patients started using the MERM. Most patients were men, had some primary or secondary school education, and lived in the Chennai metropolitan area (Table 1).

We interviewed 10 HCPs, with a median age of 35 years (range 29-54 years). They had a median of 5.5 years of work experience in the NTEP (range 2-15 years). Most HCPs were men, had an undergraduate education, and had jobs as health visitors (Table 2).

**Table 1:** Descriptive statistics for patients with multidrug-resistant tuberculosis being monitored with the Medication Event Reminder Monitor.

Characteristic		Values, n (%)
**Location**		
	Chennai	40 (62)
	Mumbai	25 (38)
**Gender**		
	Male	42 (65)
	Female	23 (35)
**Educational attainment**		
	No formal education or low literacy	13 (20)
	Some primary or secondary education	44 (68)
	Some college education, including degree or diploma holders	8 (12)
**Occupation**		
	Unemployed	16 (25)
	Student	7 (11)
	Homemaker	7 (11)
	Formal government or private sector job	6 (9)
	Self-employed	29 (45)

**Table 2:** Descriptive statistics for health care providers who were interviewed to understand their acceptance of the Medication Event Reminder Monitor.

Characteristic			Values, n (%)
**Location**			
	Chennai	5 (50)	
	Mumbai	5 (50)	
**Gender**			
	Male	6 (60)	
	Female	4 (40)	
**Educational attainment**			
	Undergraduate college education only	8 (80)	
	Postgraduate education	2 (20)	
**Designated position**			
	TB^a^ health visitor	5 (50)	
	Senior treatment supervisor	2 (20)	
	Treatment coordinator	1 (10)	
	Deputy director of TB programs	1 (10)	
	District TB officer	1 (10)	

^a^TB: tuberculosis.

### Findings From Patients With MDR-TB

**Figure 2:**
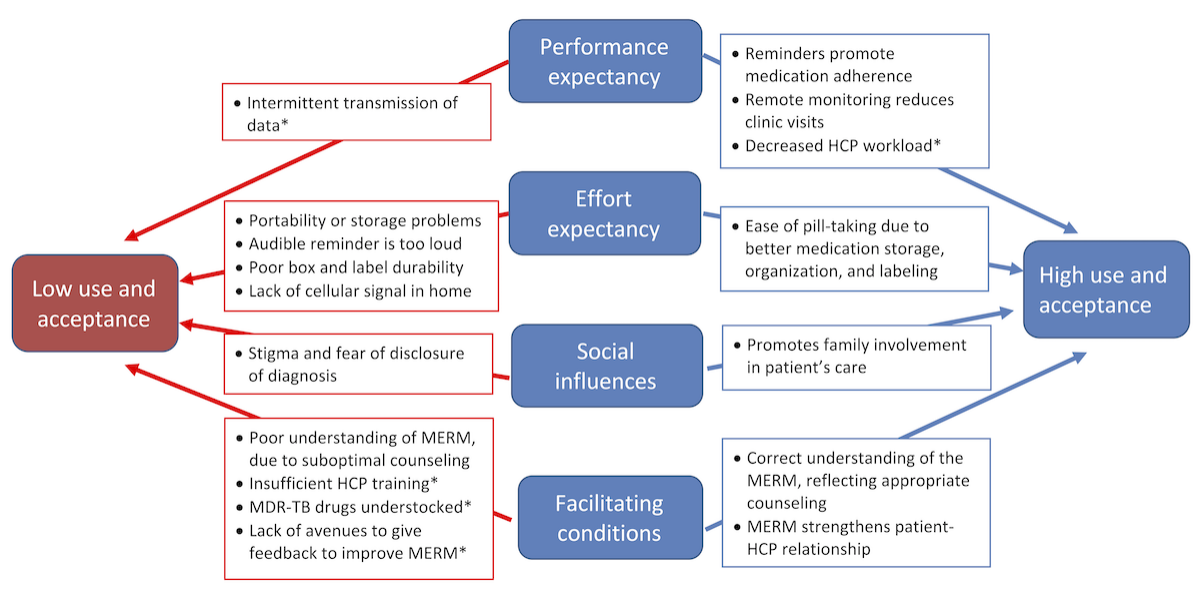
Key findings regarding determinants of high and low acceptance and use of the Medication Event Reminder Monitor by patients with MDR-TB and health care providers based on the Unified Theory of Acceptance and Use of Technology (UTAUT) framework. *Findings are from health care provider interviews; all other findings are from patient interviews. HCP: health care provider; MERM: Medication Event Reminder Monitor; MDR-TB: multidrug-resistant tuberculosis.

Interview findings revealed facilitators of and barriers to patient acceptance of MERM (Figure 2).

#### Facilitators of Patient Acceptance and Use of the MERM

Several factors were associated with a higher acceptance of MERM (Textbox 1).

With regard to performance expectancy (perceived usefulness), many patients felt that the reminders prevented them from forgetting to take their medications and helped them take it at the same time every day, with most preferring audible (Textbox 1; quote 1) and a few preferring visual (quote 2) reminders. One patient described the following benefits of audible reminders:

I finish my breakfast before 10 O’ clock and wait for the alarm to ring. The alarm is useful because even when I forget it reminds me to take the tablets.

Patient, male, 49 years

Some patients also appreciated the yellow light, which served as a reminder to return to the clinic for medication refills.

Before being given their medications in the MERM, patients with MDR-TB usually visited clinics more frequently (eg, daily or weekly) for closer monitoring by HCPs. Some patients appreciated that remote monitoring of adherence resulted in reduced time and money spent on clinic visits (quote 3), as described by the following patient:

I previously had to visit the hospital three or four times in a month, but now I am going there once a month, so it is very good that you have provided this box. It is like a blessing for me.

Patient, male, 39 years

Several patients also appreciated the manner in which the MERM stores, organizes, and provides helpful internal labeling of medications. Patients previously stored medication blisters in plastic bags or a cardboard box provided by the TB program that did not have internal partitions to organize medications (quotes 4 and 5). These findings speak both to favorable perceived usefulness (ie, performance expectancy) of the MERM for organizing medications and favorable ease of use (ie, effort expectancy) because most patients found it easy to follow and understand the MERM’s internal labels that guide pill taking, as noted by the following patient:

There are different compartments for each tablet, so they don’t get mixed with each other....It is helpful. I like the arrows with the dots that explain how many of each medication I need to take.

Patient, male, 38 years

With regard to social influences, several patients reported that the audible reminder function promoted increased involvement of family members in their TB care (quotes 6-8), although occasionally such involvement was because of annoyance from the audible reminder (quote 7) or perceptions that the MERM facilitated government surveillance (quote 8). In some cases, however, this family involvement was prompted by the positive perception that the MERM represents an extension of the care provided by HCPs:

As soon as the alarm rings, my son immediately runs to me and says “Your doctor is calling you. Go and take your medicine and then do your work.”

Patient, male, 49 years

Counseling of patients by HCPs in the appropriate use of the MERM is an important facilitating condition. The quality of counseling was assessed indirectly based on whether patients had appropriate or inappropriate knowledge of the functions of the MERM. There was variability in patient understanding of the MERM; however, most patients expressed a correct understanding of its medication labeling (quote 9) and other basic functions. For example, the following patient correctly interpreted the different lights on the MERM, reflecting appropriate counseling:

If red color [light] blinks there is no charge; if the green color blinks, it signals that the tablets have to be taken at 10 O’ clock; and if the yellow color blinks, it means that the tablets are going to run out.

Patient, male, 22 years

Some patients reported that HCPs deployed the MERM in a manner that strengthened the patient-HCP relationship (quote 10). These findings highlight aspects of the MERM’s perceived usefulness (performance expectancy), as well as favorable facilitating conditions in the health system’s implementation of this technology. For example, patients appreciated that HCPs seemed to use the adherence data generated by the MERM in a positive manner, which resulted in patients feeling *cared* *for* remotely:

If I don’t open the [pill]box, somebody from the health center calls me to find out whether I have taken the tablets or not. They care for me.

Patient, male, 61 years

**Representative quotations on factors facilitating the acceptance of the Medication Event Reminder Monitor by patients based on constructs in the Unified Theory of Acceptance and Use of Technology (UTAUT).**

**Performance expectancy**

- Reminders promote medication adherence
  Quote 1: “The sound of the alarm forces me to take the medicine on time.” (Patient, female, aged 42 years)
  Quote 2: “Even when the alarm is not audible, the light is useful, especially when I am not near the box.” (Patient, male, aged 25 years old)
- Remote monitoring reduces clinic visits
  Quote 3: “I can do work at home properly now and do not have to worry about going to the health center.” (Patient, female, aged 27 years)

**Effort expectancy**

- Ease of pill taking because of better medication storage, organization, and labeling
  Quote 4: “Previously, I kept the tablets in a plastic cover, but now they are safer in the box. I used to be so confused, as there were so many medicines to take. Now it is easier.” (Patient, female, aged 21 years)
  Quote 5: “The pills were previously given in an ordinary cardboard tablet box, which does not have an alarm, but this box has an alarm to remind me.” (Patient, male, aged 61 years old)

**Social influences**

- Promotes family involvement in patient’s care
  Quote 6: “When the alarm rings and I am outside my house, they send a person to inform me to take my pills.” [Patient, male, aged 49 years]
  Quote 7: “My mother complains when I delay taking the medicines. She would say, ‘The box has been making noise constantly’ and makes sure I take the medicines so the noise will stop.” (Patient, male, aged 44 years)
  Quote 8: “There is a camera in the box, so if you don’t take the pills, people in Delhi will come to know. So take your pills.” (Mother of a 25-year-old male patient)

**Facilitating conditions**

- Correct understanding of the Medication Event Reminder Monitor, reflecting appropriate counseling
  Quote 9: “I take the tablets according to the dots shaded in each column above the compartment.” (Patient, female, aged 28 years)
- Medication Event Reminder Monitor strengthens patient-health care provider relationship
  Quote 10: “At the time of discharge...[a] health worker explained the MERM box and told me about the need to take my medicines regularly and that the box would help remind me. Those words motivated and encouraged me. My anxiety was reduced, and I was filled with happiness.” (Patient, male, aged 53 years)

#### Barriers to Patient Acceptance and Use of the MERM

Patients also experienced barriers to the acceptance and use of MERM (Textbox 2).

A few patients admitted a lack of understanding regarding the purpose of the MERM, which suggests limitations in performance expectancy (perceived usefulness) for this minority of individuals:

I did not know that when I don’t take pills, it will be shown [to HCPs] by a computer.

Patient, female, aged 45 years

More commonly, patients described limitations in effort expectancy (ease of use). For example, one patient described how the MERM’s lack of portability led him to remove medication blisters from the device, which would result in the MERM recording adherence inaccurately:

I take my pills out of the box when I leave for work and put them in my pocket. I cannot carry such a big box to work that makes so much noise when I open it. I take the medicines [at work] when I am free. I do not benefit from the alarm or the light [audible and visual reminders] because I leave the box at home.

Patient, male, aged 21 years

Other patients similarly described how the device’s large size served as a barrier to taking it to work (Textbox 2; quote 11) or storing it inside the house (quote 12). In addition, some of these concerns were related to stigma and privacy. Patients were concerned that the device’s size and the loud sound of the audible reminder would draw attention and potentially raise questions about the patient’s underlying medication condition (quote 13). Some patients found the audible reminder to be a major annoyance (quote 14). For example, one patient propped the box open to prevent the alarm from going off:

The alarm is too loud. So to avoid it [from going off], I put a paper in between the box and the lid and take the medicine.

Patient, female, aged 18 years

This action breaks the magnetic seal on the lid of the MERM and interferes with the recording of daily dose taking, thereby resulting in an inaccurate adherence record.

Patients also found that the body of the MERM, which is made of commercial-grade cardboard, poorly withstood the humidity and monsoon weather conditions in India. Medication labels peeled from the box (quote 15), and the box’s shape became distorted:

My box bulged after it had rained continuously, and the inside of the house became damp, so it would be better if the box was made out of plastic.

Patient male, aged 48 years

A few patients found the MERM medication labels to be challenging; however, some of these difficulties may have reflected poor organization of medications in the box by HCPs, such that medications were not in the partitions corresponding to the appropriate labels:

This box is useful as there are instructions about its use, but sometimes the arrows [labeling each medication] don’t match with the [correct] medicines. I get confused.

Patient, male, aged 40 years

Patients reported various other technical problems that limited the ease of using the MERM, including weak cellular signal in the patient’s home resulting in the nonreporting of doses taken (quote 16), failure of the device’s battery (quote 17), and malfunction of the reminder lights (quote 18).

With regard to social influences, several patients reported concerns regarding violations of the privacy and confidentiality of their MDR-TB diagnoses, which reflects the stigma surrounding this disease. As described earlier, patients were particularly concerned about stigma when traveling with the MERM (quote 13), but many patients were equally concerned about stigma when taking the device with them to clinic visits (quote 19), when friends or relatives visited the home (quote 20), or when family members heard the audible reminder, even if the device was hidden (quote 21). One patient was even concerned that the audible reminder was loud enough to draw the attention of her neighbors:

When the alarm rings, my neighbors can hear it. I am scared that they will come to know about my disease.

Patient, female, aged 21 years

With regard to facilitating conditions, some patients conveyed an incorrect understanding of the functions of the MERM (quote 22). For example, when asked about the visual reminders, the following patient conveyed an incorrect understanding:

I have to close the box when the yellow light blinks and I understand that if the red light blinks the tablets are going to be over.

Patient, female, aged 75 years

**Representative quotations on barriers to acceptance and use of the Medication Event Reminder Monitor by patients based on constructs in the Unified Theory of Acceptance and Use of Technology (UTAUT).**

**Effort expectancy**

- Size, portability, and storage problems
  Quote 11: “Sometimes I have to go for work for 2 or 3 days, and during that time I can’t carry this big box to the workplace. A smaller box with an alarm would be useful when I go for work.” (Patient, male, aged 41)
  Quote 12: “I keep my box in a hen cage [outside of the house], because my children used to play with it. I don’t have a place in my home to keep the box where my children won’t reach it.” (Patient, male, aged 33 years)
  Quote 13: “How can I carry this big box when I have to attend a marriage function in my village? I am sure my relatives will ask me questions when I take the medicines out of the box and when they hear the alarm sound. I usually take the medicines out of the box when I travel and leave the box at home.” (Patient, female, aged 45 years old)
- Audible reminder is too loud
  Quote 14: “The sound is so loud, even the neighbors can hear it....Maybe it [the audible reminder] is useful for elders but not for youngsters like me because I feel irritated when it alerts me.” (Patient, female, aged 18 years)
- Limited durability of the box and labels
  Quote 15: “The label in the box is not properly fixed and it has started peeling off.” (Patient, female, aged 18 years)
- Other technical problems with the Medication Event Reminder Monitor
  Quote 16: “Sometimes due to [cellular] signal problems, although I was opening the box, these doses were not reported. I received calls from the health center [in which HCPs told me] to keep the box [at locations in the house] where the network might be better.” (Patient, male, aged 38 years)
  Quote 17: “The alarm did not ring once and when I took it to the centre, they told that the box has run out of charge and needs to be replaced or recharged.” (Patient, male, aged 44 years)
  Quote 18: “I am confused because all the three lights were glowing every day.” (Patient, female, aged 37 years)

**Social influences**

- Problems related to privacy and stigma
  Quote 19: “When I carry the box when leaving the health center, people know that I have TB. This is embarrassing, so I try to hide it, but it is too big.” (Patient, male, aged 39 years)
  Quote 20: “Suppose that my relatives visit my home. The box’s alarm could ring in front of everybody....They may come to know that I have this disease. I would be so embarrassed in front of them. So, I don’t like this box.” (Patient, female, aged 18 years)
  Quote 21: “I keep the MERM inside the cupboard in my bedroom. I go inside my bedroom and take the medicine [privately]. If the alarm goes off and there is somebody at home, they sometimes ask me where that sound came from.” (Patient, male, aged 49 years)

**Facilitating conditions**

- Incorrect understanding of the Medication Event Reminder Monitor, reflecting suboptimal counseling
  Quote 22: “The green color light helps me as a reminder but the red color means danger, which indicates that I have to go for the refill.” (Patient, female, aged 18 years old; description reflects incorrect understanding of the meaning of each light)

### Findings From HCPs

The interview findings revealed both facilitators of and barriers to HCP acceptance of the MERM (Figure 2).

#### Facilitators of HCP Acceptance and Use of the MERM

For HCPs, perceptions of positive performance expectancy (ie, perceived usefulness) were the strongest facilitators of their acceptance and use of the MERM. In particular, most HCPs felt that remote monitoring of adherence was beneficial for both patients and HCPs. During the pilot implementation of the MERM, patients with MDR-TB were generally dispensed 1 month of medications. Clinic visits to pick up medications, which were previously required on a weekly or biweekly basis, were reduced substantially under the assumption that remote monitoring minimized the need for more frequent in-person monitoring. Most HCPs felt that patients benefited from this reduction in required clinic visits, as described in the following quotation:

Patients now do not have to travel long distances, spending their money to collect their drugs every week, or sometimes twice a week. Most of our MDR TB patients come from distant villages, and transportation is very difficult. We feel comfortable giving them a month’s supply in the box [MERM] as it is easier for them to take and the light and alarm [reminders] help them to take their drugs on time.

Senior treatment supervisor

Reduced frequency of patient visits decreased the workload for many HCPs, resulting in decreased stress:

Previously, we had to supervise therapy on a daily basis [ie, DOT]. But now the patients come [to the clinic] only once a month, so our work pressure has reduced.

Senior treatment supervisor

Some HCPs reported that the decreased workload allowed them to focus more on each patient interaction, as well as other tasks, which increased job satisfaction:

I have more time now to check whether patients have taken their tablets or not. I am also able to concentrate on other tasks as well, which gives me more satisfaction in my work.

Health visitors

With regard to social influences, some HCPs perceived that providing medications in the MERM, compared with the cardboard box previously used to dispense medications, was potentially less stigmatizing for patients because some cardboard boxes contained messages regarding TB:

The good thing about the MERM is that it does not carry any messages on TB [on the outside of the box], so there is no stigma attached to it. Patients can carry it freely.

Medical officer

#### Barriers to HCP Acceptance and Use of the MERM

HCPs also reported barriers to the acceptance of the MERM for both patients and HCPs. With regard to performance expectancy, HCPs found intermittent (every 72 h) updating of patients’ adherence records to be the most significant limitation to the MERM’s perceived usefulness, as described by the following HCP:

It takes 72 hours for the [MERM] dashboard to show that the patient has taken medications. This makes it difficult for us to monitor the patient’s drug intake on a daily basis. We cannot take action as promptly and lose time.

Pharmacist

With regard to effort expectancy (ease of use), many HCPs felt that the size of the MERM made transporting the device to clinic visits prohibitive:

It is good [for patients] to have a device like the MERM, but...it is difficult to carry, as they need to go by bus and train. We need to provide them with a big bag for [the device].

Health visitors

The MERM’s size also resulted in challenges for HCPs themselves:

Even for us [HCPs] at the health centers, it is difficult to find space to store these MERM boxes.

Senior treatment supervisor

Consistent with findings from the patient interviews, HCPs described a lack of cellular signal in patients’ homes as a barrier to MERM use for rural patients in particular. This barrier contributed to limitations in HCPs’ ability to obtain adherence data from and communicate with patients:

Some of the patients are not willing to use the box [MERM], as people living in the villages are not always getting [adequate cellular] signal, so the device is not working.

Senior treatment supervisor

During the pilot implementation of the MERM, HCPs found that some facilitating conditions on the part of the health system were suboptimal. For example, some HCPs felt that the single-day training provided would be insufficient for new personnel:

One day of training will be difficult if we have newly recruited staff, because they have to understand the [MDR TB] program, and then undergo training [in use of the MERM].

Senior treatment supervisor

Some barriers to implementation arose from more fundamental challenges in the MDR-TB program. For example, MDR-TB medications were supposed to be dispensed in the MERM on a monthly basis; however, some medications were sometimes understocked. This problem was easier to manage when patients were refilling medications on a weekly or more frequent basis because fewer medications had to be dispensed at any given visit. MERM implementation therefore worsened challenges related to the understocking of drugs:

Sometimes MDR-TB drugs are not available, and so we are not able to give all the medicines required....How do we leave that compartment [in the MERM for a specific medication] empty, and what can we tell the patient?

Pharmacist

Finally, some personnel felt that when problems were identified with the implementation of the MERM, they did not have channels to communicate these challenges:

When we started using the MERM, we were excited about the device. When patients came back for their medication refills, they raised concerns with regard to technical problems—the alarm, light, texture and size of the box, for example. I was not sure who to notify. Maybe we could have had those who made the device discuss our feedback so it could be improved?

Senior treatment officer

## Discussion

### Principal Findings

This study describes the evaluation of a low-cost digital pillbox aimed at promoting medication adherence among patients with MDR-TB during pilot implementation in India’s NTEP. We find that acceptability of the MERM is variable; some features of the technology facilitate acceptability, whereas other features and contextual factors serve as barriers to engagement by some patients. Although previous studies have evaluated the use of similar digital pillboxes as part of TB care [3,18-20,22,23], to our knowledge, only one previous study conducted in South Africa [17] has evaluated the use of these technologies for patients with MDR-TB, who face unique challenges, including the complexity of their medication regimens, prolonged duration of therapy, increased risk of drug toxicities, and greater disease-related stigma. In addition, our study is unique in that it assessed the perspectives of both patients and HCPs.

### Implications of Findings From Patients With MDR-TB

Multiple factors increase patient acceptance and the use of MERM. For most patients, audible and visual signals served as helpful reminders to take medications. Forgetfulness is a common barrier to adherence [36]. Although often thought of as a cognitive problem, forgetfulness may also reflect other life challenges faced by patients, such as depression or spending long hours at work. In a qualitative study in India assessing the acceptance of 99DOTS, a cellphone-based DAT used to monitor patients with drug-susceptible TB, most patients reported that SMS text messages were not useful reminders to take medications because these messages were lost amid a high volume of *spam* SMS text messages [35]. In contrast, an advantage of the MERM was that the reminders drew patients to the site where medications were stored. This increased the likelihood that patients with MDR-TB immediately took their doses, which may promote habit formation in pill taking behavior [37,38]. In addition, the MERM’s reminders sometimes transformed household social dynamics by drawing family members into patients’ TB care, a finding also reported in studies of other DATs [35].

Patients appreciated aspects of the MERM’s design—in particular, the secure storage provided by the box, the labels to help patients take an appropriate number of tablets of each medication, and the organization of different medications facilitated by internal partitions. These features were valued in light of the complexity of MDR-TB treatment regimens, which include at least 4 or 5 different medications, as well as the fact that MDR-TB medications had previously been dispensed in a cardboard box without internal partitions to separate medications or labels to guide pill taking.

In some patients, MERM enhanced their relationship with the health system. Most patients appreciated saving time and money by not having to visit the clinic as often because the frequency of routine clinic visits for patients with MDR-TB was reduced during the MERM pilot implementation. Although this resulted in decreased face-to-face interactions with HCPs, some patients actually described feeling more *cared for*. This feeling was derived from the perception that HCPs were remotely watching over their clinical progress, as well as from positive responses to actual phone or in-person outreach by HCPs, guided by patients’ adherence data. Previous studies evaluating the use of DATs to support HIV and TB treatment adherence in Uganda, India, and South Africa similarly found that remote monitoring enhanced some patients’ perceptions of the care provided by the health system [17,35]. This may be one of the behavioral pathways by which DATs may motivate patients to adhere to treatment.

Patients also reported barriers to the acceptance and use of MERM. Some of these barriers may be modified by altering the MERM’s design or implementation (Textbox 3). For example, the loud volume of the audible reminder—a common complaint from patients also reported in a previous study of the MERM from China [18]—could potentially be modified or the audible reminder disabled completely, ideally based on patients’ personal preferences. As another example, during pilot implementation, the MERM was made of commercial-grade cardboard, which did not wear well in India’s humid weather conditions. Redesigning the MERM using plastic would be feasible and minimize weather-related damage, although it would likely increase the cost of the device. Other technical problems, such as battery failure or inappropriate blinking of the reminder lights, could likely be addressed with product improvements in future iterations of the MERM.

**Recommendations for improving the Medication Event Reminder Monitor device and its implementation, based on findings from patients with multidrug-resistant tuberculosis and health care provider interviews.**

**Design of the device**

- Data transmission from the device on a daily basis may facilitate better near real-time monitoring
- Redesign using plastic (rather than cardboard) may reduce wear because of weather conditions
- Strengthening internal partitions may help avoid accidental mixing of different medications
- Device reuse should be limited, given considerable wear and tear even after single patient use

**Reminder functions**

- Allowing patients to reduce the volume of the audible reminder or to deactivate audible or visual reminders may address concerns about privacy and stigma
- Malfunction of visual reminders (eg, all lights blinking at once) should be fixed

**Counseling and monitoring of patients**

- Health care providers should be trained to provide standardized counseling to ensure patient understanding of key Medication Event Reminder Monitor functions
- Health care providers should use pill counts and ask adherence questions to patients at clinic and home visits to cross-check the Medication Event Reminder Monitor’s adherence data

**Screening out patients for whom the Medication Event Reminder Monitor may not be appropriate**

- Systematic screening should be performed upfront to identify patients for whom the Medication Event Reminder Monitor may not be appropriate, including those with concerns about stigma, a fear of disclosure of diagnosis, difficulties with portability, and lack of cellular signal in the home

**Training of health care providers**

- Mechanisms should be created for the training of newly hired National Tuberculosis Elimination Program personnel and provision of periodic refresher training in the Medication Event Reminder Monitor for existing personnel
- Mechanisms should be created for National Tuberculosis Elimination Program personnel to provide ongoing feedback to facilitate device improvements

Although some barriers may be addressable, others may present more fundamental challenges that could limit the use of the MERM among some patients. For example, the MERM’s large size was a barrier for patients who were traveling or who preferred to take their medications at work. However, the MERM’s size is necessary to hold a 1-month supply of MDR-TB medications, and patients benefit from having their medications dispensed in an organized manner with appropriate labeling. Furthermore, because of its prohibitive size, patients who need to take doses when traveling or at work tend to remove doses from the device rather than carry it with them. The lack of cellular signals in the home is another nonmodifiable barrier that limits the benefits of remote monitoring because adherence data cannot be transmitted from the device on a regular basis.

Disease-related stigma, from family and community members, is a common challenge faced by patients with MDR-TB [10]. Owing to stigma, patients often do not disclose their diagnosis to family members, friends, and coworkers; as a result, patients fear situations that could result in disclosure of their diagnosis to others. The MERM’s large size, as well as its audible and visual reminders, raised patient concerns regarding the risk of disclosure of diagnosis. Indeed, as ascertained from their interviews, patients who faced barriers related to their social context, including stigma, a fear of disclosure, or frequent work-related travel, seemed to be the most likely to not use the MERM.

All of these problems—removal of doses from the device because of its lack of portability, the nonreporting of box openings because of lack of cellular signal, and the nonuse of the device because of disease-related stigma—could result in underreporting of medication doses, resulting in inaccuracies in patients’ adherence records. Recent studies found that these same barriers contribute to relatively high rates of patient nonengagement with 99DOTS in India [35], especially in the continuation phase of therapy, which contributed to the technology’s suboptimal accuracy for measuring adherence to TB medications [24]. A qualitative study of drug-susceptible TB patients monitored with the MERM in Vietnam found that only approximately half of the patients used the device as intended largely because of the difficulties with the device’s portability, with the result that the MERM data often did not reflect actual adherence [23]. A high rate of device nonuse was also found in a study that used the Wisepill device (a similar digital pillbox) to monitor adherence to HIV preexposure prophylaxis in young men who have sex with men in the United States [39].

These barriers to use suggest that if implementation of the MERM is expanded among patients with MDR-TB in India, there could be limits to the device’s reach, or overall coverage, in this patient population. Wide-scale implementation of a similar digital pillbox among patients with drug-susceptible TB in China has revealed meaningful limitations in the reach of the device [21,22]. For example, in one study of the implementation of a digital pillbox in 30 counties in China, even after excluding 41% of the patient cohort who were either not eligible to use the device or who did not receive the device for unclear reasons, only approximately two-thirds of the remaining 1314 patients who received the pillbox had sustained use for the remainder of the treatment [22]. The other one-third of patients who received the digital pillbox either stopped using the device or met the criteria to be shifted back to monitoring with DOT because of a high proportion of missed doses. These missed doses could have represented either true nonadherence to medications or inappropriate use of the device.

In light of such findings from other contexts, it would be reasonable to assume that some proportion of patients with MDR-TB in India might not use the MERM in wide-scale implementation. On the basis of our findings, the NTEP could consider screening patients with MDR-TB upfront to identify individuals who might be unlikely to use the device, for example, because of patient concerns about stigma and portability or lack of cellular signal in the home (Textbox 3). In addition, HCPs should use other strategies to verify medication adherence, including pill counts and asking adherence-related questions to patients at every in-person clinic and home visit, which will help HCPs to cross-check the adherence data being received from the MERM.

### Implications of Findings From HCPs

In the HCP interviews, NTEP personnel affirmed some of the patient-oriented benefits of the MERM, in particular, the time and money saved by patients from the reduced frequency of clinic visits; however, HCPs’ perceptions that the MERM was associated with fewer patient concerns about stigma were not shared by some patients. HCPs also reported that implementation of the MERM reduced their workload because of the reduced frequency of clinic visits by patients and the ability to monitor adherence from the clinic rather than by home visits. As a result, HCPs dedicated greater time to other tasks and reported improved job satisfaction, similar to the findings of a previous study of the MERM conducted in China [18]. HCPs did find some aspects of the pilot implementation to be suboptimal; however, most of these concerns were potentially addressable. In particular, they reported a need for more training in the use of the MERM, especially in light of the high turnover of staff, and the need for a platform to communicate any implementation challenges they faced (Textbox 3).

### Directions for Future Research

This initial evaluation has identified several features that may facilitate high acceptability of the MERM for many patients with MDR-TB, especially if modifications are made to improve the device. Future research should focus on understanding how often patients face critical barriers to acceptability (eg, disease-related stigma), the extent to which these barriers lead to device nonuse and whether screening for these barriers can be used to better target the MERM to patients most likely to use it. In addition, further research is needed to understand the accuracy of the MERM for measuring adherence to MDR-TB medications, its effectiveness for improving treatment outcomes, and its reach, that is, coverage or uptake by patients, in large-scale implementation [3].

Even for patients who agree to use the MERM, the adherence record could be inaccurate because of underreporting (eg, if patients take medications out of the device, resulting in device nonuse) or overreporting (eg, if patients open and close the device without actually taking medications). Indeed, a recent study of 99DOTS, in which its adherence record was compared with urine isoniazid test results from patients with TB collected during unannounced home visits, found that both under- and overreporting of adherence contributed to the technology’s suboptimal accuracy [24]. A similar research approach, involving unannounced home visits with measurement of urine biomarkers for MDR-TB medications, could be used to evaluate the accuracy of MERM, although pill counts should also be conducted to provide insights into whether patients have differential adherence to different medications in the MDR-TB regimen.

Existing studies of the use of DATs to promote adherence to TB medications have found both positive [20,40] and negative or equivocal [41-43] impacts on adherence and TB treatment outcomes. As such, studies of effectiveness, especially high-quality randomized trials, are needed to assess whether MERM use translates into improvements in treatment outcomes and recurrence-free survival for patients with MDR-TB. Even when DATs have been shown to be effective, as with digital pillboxes in China [20], subsequent large-scale implementation studies have shown suboptimal reach or coverage of patients [21,22]. As such, studies of the MERM’s coverage of patients with MDR-TB in large-scale implementation will be critical to ensure that it achieves population-level impact. Finally, in light of the diverse psychosocial barriers to adherence faced by patients with MDR-TB [10], the benefits of monitoring with the MERM in this population will depend on the development of interventions to address problems, such as medication toxicities, depression, stigma, and substance use disorder, which are often the underlying causes of nonadherence [44].

### Study Limitations

Our study was limited to assessing patient and HCP perceptions, rather than more objective findings, such as the accuracy of the MERM or impact on clinical outcomes. As such, we may have overestimated the acceptability and benefits of this technology because of socially desirable responses, which is a common bias in qualitative research. In addition, patients attributed the reduced frequency of their clinic visits to the MERM, as a longer supply of medications was dispensed in the device. The reduced frequency of clinic visits may have therefore biased patients in favor of higher acceptance of the device; however, provision of a longer supply of medications could have just as easily been implemented without the MERM.

Our deductive approach to analysis allowed us to organize and report our findings using the UTAUT, which is a robust and evidence-based framework for understanding technology acceptance; however, a limitation of this approach is that we could have overlooked findings that did not fit into this predetermined framework.

Another limitation of our study is that we assessed patients’ perceptions of the MERM within a few weeks of their use of the device. In light of the prolonged duration of MDR-TB treatment, it is possible that patients’ acceptance and use of the device could change over time. In addition, our study was limited to 2 cities and may not be representative of barriers to the use of the MERM in rural parts of India. Future studies could consider including diverse geographic settings and conducting multiple interviews to understand the acceptability of the MERM throughout the treatment course.

### Conclusions

In this study of the pilot implementation of a low-cost digital pillbox to promote adherence to MDR-TB medications, we identified several features that facilitate high acceptability of the device among patients. These included helpful organization and labeling of medications, feeling more *cared for* by the health system because of remote monitoring, and appreciation of the audible and visual reminders, which often drew family members into patients’ care.

At the same time, we identified barriers that could limit the acceptance and use of the MERM by some patients. Although some of these barriers could be addressed relatively easily with modification of the device, other barriers—such as difficulties with the device’s portability, lack of cellular signal in the home, and a fear of disclosure of diagnosis because of disease-related stigma—are more difficult to modify and may limit the reach or population coverage of this technology. Future research is needed to assess the accuracy of the MERM for measuring adherence, its effectiveness for improving treatment outcomes, and patients’ sustained use of the device in larger scale implementation in India’s MDR-TB treatment program.

## Appendix

Multimedia Appendix 1 Examples of questions included in the in-depth interview guide for patients with multidrug-resistant tuberculosis in relation to constructs in the Unified Theory of Acceptance and Use of Technology (UTAUT).

## Abbreviations

DAT: digital adherence technology
DOT: directly observed therapy
HCP: health care provider
MDR-TB: multidrug-resistant tuberculosis
MERM: Medication Event Reminder Monitor
NTEP: National Tuberculosis Elimination Program
TB: tuberculosis
UTAUT: Unified Theory of Acceptance and Use of Technology
