# Supplementary material for: Acceptability of the Medication Event Reminder Monitor for Promoting Adherence to Multidrug-Resistant Tuberculosis Therapy in Two Indian Cities: Qualitative Study of Patients and Health Care Providers
Source: J Med Internet Res. 2021 Jun 10;23(6):e23294. doi: 10.2196/23294 (PMC8262665; doi:10.2196/23294)
Supplement: Multimedia Appendix 1 [file jmir_v23i6e23294_app1.doc]

**Multimedia Appendix 1. Examples of questions included in the in-depth interview guide for MDR TB patients in relation to constructs in UTAUT**

| **Performance expectancy (ie, perceived usefulness)** |
| --- |
| How do you think the digital pillbox works? (including follow-up probes to assess understanding of the reminder function, recording of the dosing history, etc) |
| Do you find the digital pillbox to be useful for your TB care? If so, why? If not, why not? |
| Is the pillbox helpful for safely storing your medications? If so, why? If not, why not? |
| Are the reminders from your digital pillbox helpful in reminding you to take your TB medications? If so, why? If not, why not? |
| Do you think the electronic pillbox is helpful to your TB doctors and care providers? If so, how? If not, why not? |
| How do you think that the digital pillbox will be useful for other TB patients in future? |
| **Effort expectancy (ie, ease of use)** |
| After receiving counselling about the digital pillbox, what have you found difficult to understand about using the digital pillbox? |
| Did you face any difficulties when you went home and first started to use your digital pillbox to help you take your TB pills? If yes, what kind of difficulties did you face? If not, what made the digital pillbox easy to use? |
| Do you always store your medications in the digital pillbox? Or have you taken your pills out of the pillbox for extended periods of time? If so, why? |
| **Social influences** |
| Does your family know about the digital pillbox? If yes, what do they think about it? |
| Has the digital pillbox changed the involvement of your family in your TB care? |
| Do you have any concerns about maintaining the confidentiality of your TB diagnosis because of the visibility of this pillbox? |
| Do you have any concerns about maintaining the confidentiality of your TB diagnosis due to audio and glowing light reminders? |
| **Facilitating conditions (ie, health system’s organizational infrastructure)** |
| When you were given the digital pillbox, was the counselling clear and understandable? |
| Have your TB doctors or other care providers used information from the digital pillbox to counsel you about how you are taking you medications? |
| Because of information from the digital pillbox, have your TB doctors or other care providers visited your home to provide counseling about how you are taking your TB medications? |
| How do you feel the digital pillbox affects your relationship with your TB doctors and the TB program? |
